# A Novel *SERPINA1* Mutation Causing Serum Alpha_1_-Antitrypsin Deficiency

**DOI:** 10.1371/journal.pone.0051762

**Published:** 2012-12-12

**Authors:** Darren N. Saunders, Elizabeth A. Tindall, Robert F. Shearer, Jacquelyn Roberson, Amy Decker, Jean Amos Wilson, Vanessa M. Hayes

**Affiliations:** 1 Cancer Research Program, Garvan Institute of Medical Research, Sydney, Australia; 2 Kinghorn Cancer Centre, Sydney, Australia; 3 St Vincent’s Clinical School, Faculty of Medicine, University of New South Wales, Sydney, Australia; 4 Medical Genetics, Henry Ford Hospital, Detroit, Michigan, United States of America; 5 Quest Diagnostics Nichols Institute, Valencia, California, United States of America; University of Giessen Lung Center, Germany

## Abstract

Mutations in the *SERPINA1* gene can cause deficiency in the circulating serine protease inhibitor α_1_-Antitrypsin (α_1_AT). α_1_AT deficiency is the major contributor to pulmonary emphysema and liver disease in persons of European ancestry, with a prevalence of 1 in 2500 in the USA. We present the discovery and characterization of a novel *SERPINA1* mutant from an asymptomatic Middle Eastern male with circulating α_1_AT deficiency. This 49 base pair deletion mutation (T379Δ), originally mistyped by IEF, causes a frame-shift replacement of the last sixteen α_1_AT residues and adds an extra twenty-four residues. Functional analysis showed that the mutant protein is not secreted and prone to intracellular aggregation.

## Introduction

Mutations in the *SERPINA1* (*PI*) gene can cause loss or deficiency in the circulating serine protease inhibitor, α_1_-Antitrypsin (α_1_AT). α_1_AT is primarily secreted by the liver and plays a key role in protecting the lower respiratory tract from proteolytic damage by inhibiting neutrophil elastase. Normal α_1_AT levels, resulting from two copies of the common *SERPINA1* M allele, range between 1.5 and 3.5 g/l. α_1_AT deficiency is one of the most common hereditary disorders, with an estimated incidence rate of 1 case per 2500 individuals, yet the condition remains undiagnosed in many patients [1], [2]. Clinical conditions associated with α_1_AT deficiency primarily arise from either tissue damage due to uncontrolled elastase activity in the lungs, or from accumulation of misfolded or aggregated protein in the liver [3].

The most common α_1_AT deficient variants are known as the Z^(E342K)^ and S^(E264V)^ mutants, with the Z allele being the major contributor to pulmonary emphysema and liver disease in persons of European ancestry [4]. Protein assays based on isoelectric focusing (IEF) and differing migration patterns are the predominant method for identifying *SERPINA1* ‘deficiency’ mutations.

*SERPINA1* alleles are expressed codominantly, thus the type and combination of mutations will result in varying levels of circulating α_1_AT and associated clinical manifestation. Over 100 *SERPINA1* mutations have been identified to date, at least 30 of which have been implicated in disease pathogenesis [5]. α_1_AT deficiency is best managed with early and accurate diagnosis, which presents challenges because of the polymorphic nature of this gene as well as limitations associated with IEF testing. In this study we describe a novel 49 base pair deletion of the *SERPINA1* gene in a patient presenting with deficiency of circulating α_1_AT.

## Materials and Methods

### Mutation Detection and Variant Confirmation

A previously described denaturing gradient gel electrophoresis (DGGE) method was used for screening the entire coding region and splice junction regions of the *SERPINA1* gene for DNA variants [6]. In brief, using optimal DGGE fragment selection and primer design [7], and improvements on DGGE conditions [8], all seven amplicons were screened within two gel lanes for a single individual, allowing for overnight analysis. Aberrant DGGE bands were excised from the 40% to 80% urea and formamide denaturing polyacrylamide gel, the amplified mutated fragment allowed to elute from the band overnight in distilled water before undergoing direct Sanger sequencing. Cleaned PCR products were sequenced using the non-GC-clamped primer and Big Dye Terminator chemistry on a 3100 Genetic analyzer (Applied Biosystems). This approach allows for both variant confirmation and nucleotide-specific classification.

### Ethics

This sample was obtained for clinical purposes and the requisition stated that remnant, de-identified samples could be made available for research. We did not obtain specific IRB approval for this study. However, this study is exempt from requiring ethical approval under Australia's National Health and Medical Research Council guidelines and National Statement on Ethical Conduct in Human Research (2007). Any patient information has been sufficiently anonymised so that neither the patient nor anyone else could identify the patient with certainty.

### Cloning

An ORF clone encoding wild-type SerpinA1 was obtained from the Human ORFeome library [9]. To generate the T379Δ mutant ORF we employed gene synthesis (Geneart) to generate a short fragment containing the 3′/C-terminal extension flanked by XbaI and BstXI sites and then subcloned this fragment into the wild-type clone by restriction digestion and ligation. Subcloning was verified by restriction digest and sequencing using the following primers (GGTGCCTATGATGAAGCGTT and CAGGAAACAGCTATGAC). Expression clones encoding for wild-type and mutant SerpinA1 with either N- or C-terminal EGFP fusions were generated by Gateway™ recombination cloning onto the pcDNA6.2-DEST-emGFP or pDEST47 backbones (Invitrogen) and fusion integrity was verified by sequencing with the following primers (CGCAΑATGGGCGGTAGGCGTG and CCATCTΑATTCAACAAGΑATTGGGACAAC).

### Cell Culture

HEK293T cells (grown in DMEM with 10% FBS) were seeded into 6-well plates containing glass coverslips. Media was replaced with serum-free Optimem prior to transfection with 1 µg plasmid DNA in 2 µl Lipofectamine 2000 (Invitrogen), and cells were cultured back into complete medium 24 hours post-transfection. Coverslips, lysates, and conditioned media were harvested 48 hours post-transfection. Conditioned medium (1.5 ml) was concentrated (to ∼50 µl) using Amicon Ultra-4 10 kDa centrifugal filters (Millipore). Cell lysates were prepared using RIPA buffer with Complete™ protease inhibitor cocktail (Roche).

### GFP-trap Affinity Purification

Conditioned media (500 µL) from transfected HEK293T cells was collected after 48 hrs and secreted GFP-α_1_AT fusion protein purified by immunoprecipitation using the GFP-Trap-A reagent (Chromotek) according to manufacturer’s standard protocol.

### Western Blotting and Fluorescence Microscopy

SDS-PAGE followed by western blotting was performed on cell lysates, insoluble pellets, and concentrated conditioned media (15 µg and 30 µg total protein, respectively). Blots were blocked in 5% Skim milk powder in TBS/Tween and probed with 1∶1000 anti-GFP (A11122, Invitrogen) or 1∶1000 anti-α_1_AT (ab129354, Abcam) rabbit polyclonal antibody, followed by 1∶5000 HRP-linked Donkey anti-Rabbit IgG (NA934V, GE Healthcare). Mouse anti-B-actin (A5441, Sigma Aldrich) was used as a loading control. Cells for fluorescence microscopy were grown on coverslips and prepared using Vectashield Mounting Medium containing DAPI (Vector Laboratories).

## Results

### Patient

A Middle Eastern male in his twenties presented as an asymptomatic carrier with serum α_1_AT levels in the low-carrier range of 0.58 g/l (11 µM) as measured by nephelometry, and a Z/M2 phenotype classification as measured by IEF. Attempted confirmation of α_1_AT allele status using the Invader™-based assay (Focus Diagnostics Inc., Cypress, CA) for Z and S allele detection, and targeted Sanger sequencing over the codon 342 region (extending 300 bases) suggested an incorrect IEF diagnosis.

### Identification of SERPINA1 Mutation

**Figure 1:**
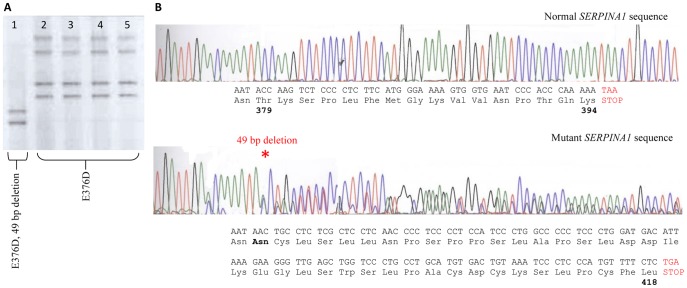
Identification of a novel SerpinA1 Mutant. A. DGGE banding patterns representing four controls (lanes 2–5) heterozygous for the M3 mutation (E376D), while our patient (lane 1), although also heterozygous for the M3 variant also presents with a shifted (faster migrating) banding depicting the novel deletion mutation (T379Δ). **B.** Sanger sequencing defines the deleted base pairs. The predicted amino acid sequence resulting from the novel 49 bp deletion (denoted by * on lower chromatogram) observed in our patient, results in the replacement of 16 amino acids and the addition of 24 amino acids through partial translation of the 3′ UTR.

Using our previously described *SERPINA1* DGGE-based variant detection method [7], we confirmed the incorrect Z/M2 diagnosis and definitively identified the patient as heterozygous for two variants; including the M3 variant (E376D) on an M1 (V213) background, and a novel 49 base deletion mutation (g.12052_12100del #K02212 genomic sequence). This deletion results in a frame-shift at position T379 that replaces the last 16 amino acids of a_1_AT and adds an additional 24 amino acids through partial translation of the 3′ UTR (Figure 1). This mutation has not previously been reported and joins the Z (E342K), S (S53F) and Mm (F52Δ) as pathogenic mutants causing profound plasma deficiency [10]. The additional amino polypeptide sequence has very little homology to any known protein sequence and hence the likely structural implications of replacing the additional residues are not immediately apparent.

### Functional Analysis: Mutant Protein Expression and Secretion

**Figure 2:**
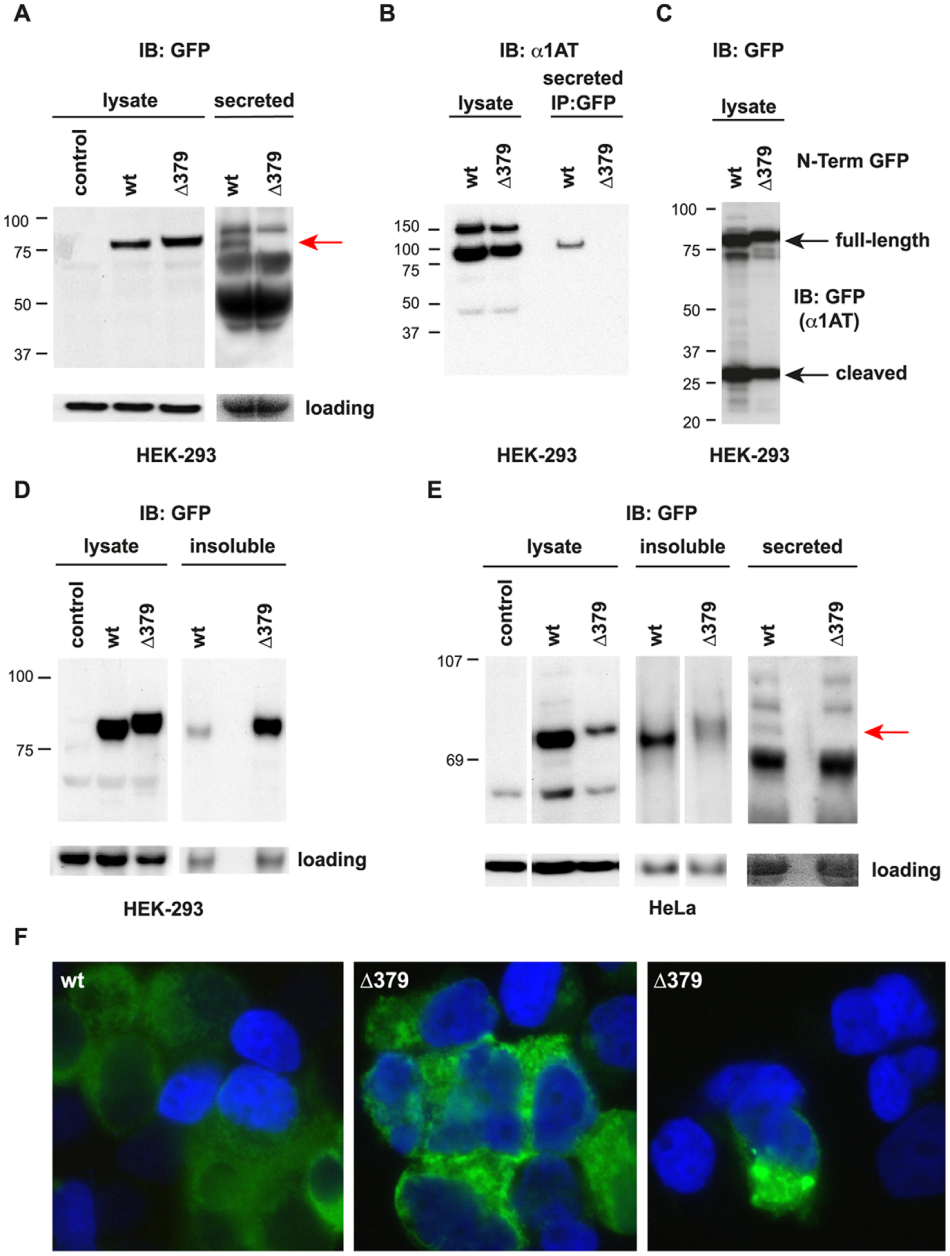
Functional Characterisation of α_1_AT ^Δ**379**^ **Mutant.** (A) Immunoblot (anti-GFP) detection of α_1_AT-GFP fusion protein (C-terminal tag) in whole-cell lysate and concentrated conditioned media (ie secreted) from HEK293T cells transfected with plasmids expressing either wild-type or Δ379 mutant α_1_AT-GFP. Red arrow denotes position of ∼75 kDa α_1_AT-GFP band, note the absence of this band in conditioned media from cells transfected with Δ379 mutant, indicating impaired secretion of mutant protein; (B) Immunoblot (anti-α_1_AT) detection of α_1_AT-GFP fusion protein (C-terminal tag) in whole-cell lysate, or following immunprecipitation from conditioned media (i.e. secreted) from HEK293T cells transfected with plasmids expressing either wild-type or Δ379 mutant α_1_AT-GFP; (C) Transfection of either wild-type or Δ379 mutant α_1_AT with an N-terminal EGFP fusion into HEK293T cells clearly indicated normal proteolytic processing of the secretion signal peptide. Both ∼75 kDA and ∼27 kDA bands are visible, representing full-length and processed (i.e. signal peptide cleaved) α_1_AT-GFP fusion protein respectively; (D) At higher expression levels, accumulation of insoluble Δ379 mutant α_1_AT was observed in HEK293T cells, clearly denoted by the presence of a darker band in the insoluble fraction from cells transfected with Δ379 mutant; (E) Detection of soluble (whole-cell lysate), insoluble and secreted (concentrated conditioned media) α_1_AT in HeLa cells transfected with either wild-type or Δ379 mutant α_1_AT-GFP. Red arrow denotes position of ∼75 kDa α_1_AT-GFP band. Note the absence of this band in conditioned media from cells transfected with Δ379 mutant, indicating impaired secretion of mutant protein; (E) Fluorescent micrographs of HEK293T cells following transfection with either wild-type or Δ379 mutant α_1_AT-GFP expression plasmids. Increased intracellular aggregation of mutant protein is clearly visible. NB: Loading controls represent α-tubulin immunoblot or PonceauS staining in lysate or secreted (conditioned media) samples, respectively.

Consistent with the clinical observation of low circulating α_1_AT levels in the patient, functional analysis showed clearly that α_1_AT^T379Δ^ is not secreted and is prone to intracellular aggregation. We observed expression of both wild-type and T379Δ α_1_AT protein in HEK293T and HeLa cell lysates following transfection (Figure 2). The slightly slower migration of the mutant form reflects the larger protein resulting from the C-terminal extension. Notably, with high-level expression in HEK293 cells there is a striking accumulation of α_1_AT^T379Δ^ in the insoluble fraction following cell lysis (Figure 2D), likely indicating misfolding and/or aggregation of the mutant form. Immunofluorescence microscopy indicated the presence of intracellular aggregates of α_1_AT^T379Δ^ in HEK293T cells (Figure 2F). Significantly, although wild-type α_1_AT is clearly detectable in conditioned media from transfected HEK293 or HeLa cells, the mutant form is not detectable (Figure 2A, D).

Impaired secretion of α_1_AT^T379Δ^ was also confirmed by performing GFP-based affinity purification of conditioned media from transfected HEK293T cells, followed by immunoblot detection of α_1_AT (Figure 2B). These experiments clearly showed secretion of wt α_1_AT, while no secretion of α_1_AT^T379Δ^ could be detected, even after GFP-trap enrichment. Cleavage of an N-terminal GFP tag from both wild-type and α_1_AT^T379Δ^ confirms normal processing of the secretion signal tag (Figure 2C) and suggests that intracellular aggregation/misfolding inhibits secretion of α_1_AT^T379Δ^.

## Discussion

A link between circulating deficiency of α_1_AT and misfolding or polymerisation of the protein has been known for over 20 years. However, despite some elegant and detailed structural analyses, the precise mechanism and exact nature of the pathogenic polymeric forms has been difficult to define. Understanding the structural and/or environmental factors driving α_1_AT misfolding are key to understanding α_1_AT deficiency and improving diagnosis and therapy.

We describe here a novel *SERPINA1* mutant from an asymptomatic patient with circulating α_1_AT deficiency. A 49 base pair deletion results in a frame-shift at amino acid T379, replacing the last 16 amino acids of α_1_AT and adding an additional 24 amino acids through partial translation of the 3′ UTR. Intracellular accumulation and failed secretion of the α_1_AT^T379Δ^ mutant in cultured cells is consistent with clinical observation of low circulating α_1_AT in the patient and establishes the mutation, along with the *Z*, *S* and *Mm* variants, as a *bone fide* pathogenic variant. Importantly, this represents the first pathogenic mutation identified in the C-terminal domain of α_1_AT, which was recently implicated in the formation of pathogenic α_1_AT polymers [11], [12]. Normal circulating levels of α_1_AT range from 104 to 276 g/L (20–53 uM). Lung disease associated with diminished neutrophil elastase inhibitory capacity is typically observed in patients with decreased circulating α_1_AT (0.36–0.57 g/L (5–11 µM)) [13]. The circulating α_1_AT level of 0.58 g/L (11 µM) observed in this patient lies at threshold of this disease-associated range.

The T379Δ mutation occurs in the C-terminal region of α_1_AT, quite distinct from the Z^(E342K)^ and S^(E264V)^ mutants found commonly in European populations but relatively rarely in African populations [6], [14]. It is noteworthy that the patient was of Middle Eastern descent, and it is highly likely that as yet unidentified deleterious α_1_AT mutations exist in other population groups that have not been well studied. Critically, these novel mutants may be missed by commonly used phenotyping approaches, further emphasizing the importance of specific genotype-based assays for accurate classification of mutants and diagnosis of α_1_AT deficiency [6], [15]. This point is highlighted by the fact that the patient in this study was originally mistyped by IEF as having a *Z/M2* phenotype classification. This study further highlights the significance of rare mutations in clinically relevant α_1_AT deficiency.

Serpins are flexible molecules capable of extreme conformational change, making them highly susceptible to polymerization. Polymer-causing mutations (such as the α_1_AT *Z* mutant) influence the folding pathway by increasing the lifetime of a polymergenic folding intermediate. Serpin polymers are favored when secondary structural domain swaps occur at a faster rate than folding into the native state. The various pathological serpin mutants identified to date have been shown to accelerate this domain swapping [11], [12]. Using a monoclonal antibody specific for hepatocellular inclusions of α_1_AT, Yakasaki *et *al [12] recently proposed a mechanism of pathological polymerization involving a C-terminal domain swap, distinct from the accepted model involving an s4As/5A swap. The implication of this observation is that the native state of α_1_AT is achieved by rapid folding of the C-terminal domain [16]. However, the exact nature of the toxic form of α_1_AT polymers in the liver is yet to be determined and may involve heterogeneous populations of polymers [11]. Although the structural consequences of the T379Δ mutation are not immediately obvious, it is highly significant that the mutation introduces an entirely new, extended C-terminal sequence into α_1_AT. This is likely to drastically modify the folding rate of the C-terminal domain of the T379Δ mutant, possibly favoring polymerization.

Interventions modifying the folding pathway of α_1_AT might be of therapeutic value in treating both loss and gain of function manifestations of α_1_AT deficiency [12]. Indeed, a number of strategies designed to attenuate polymerization are under investigation as potential therapies for α_1_AT deficiency, including peptide analogues, chemical chaperones, and small molecule allosteric regulators [13], [17], [18]. Some of these strategies (particularly peptide analogues and allosteric regulators) target specific polymerigenic mutations (e.g. Z^E342K^) and so would not necessarily be effective against the T379Δ variant. This further highlights the need to better describe the range of pathogenic α_1_AT mutations and for detailed understanding of the exact mechanisms of polymer formation.

### Conclusions

In summary, we describe a novel pathogenic *SERPINA1* mutation causing circulating α_1_AT deficiency. This mutation provides novel insight into mechanisms of α_1_AT misfolding in liver and lung disease, with important implications for molecular diagnosis and therapeutic development.
